# Immobilization of Ionophore and Surface Characterization Studies of the Titanium(III) Ion in a PVC-Membrane Sensor

**DOI:** 10.3390/s120708806

**Published:** 2012-06-27

**Authors:** Majid Rezayi, Lee Yook Heng, Anuar Kassim, Saeid Ahmadzadeh, Yadollah Abdollahi, Hossein Jahangirian

**Affiliations:** 1 School of Chemical Sciences and Food Technology, Faculty of Science and Technology, Universiti Kebangsaan Malaysia, 43600 Bangi, Selangor D.E., Malaysia; 2 ACECR Mashhad Branch, Food Science and Technology Research Institute, Mashhad 91775-1376, Iran; 3 Department of Chemistry, Faculty of Sciences, Universiti Putra Malaysia, 43400 Serdang, Selangor, Malaysia; E-Mails: anuarusj@gmail.com (A.K.); chem_ahmadzadeh@yahoo.com (S.A.); h.k@jahangirian.com (H.J.); 4 Advanced Materials and Nanotechnology Laboratory, Institute of Advanced Technology Universiti Putra Malaysia, 43400 Serdang, Selangor D.E., Malaysia; E-Mail: yadollahabdolla@putra.upm.edu.my

**Keywords:** ionophore-immobilized membrane, titanium(III) cation, c-methyl-calix[4]resorcinarene, UV-Vis, Fourier transform infrared spectroscopy, scanning electron microscopy, X-ray diffraction

## Abstract

Novel ionophores comprising various hydroxide and amine structures were immobilized onto poly(vinyl chloride) (PVC) matrices, and these were examined to determine Ti(III) selectivity. To predict the selectivity of Ti(III), a PVC membrane was used to investigate the binding of Ti(III) to c-methylcalix[4]resorcinarene (CMCR). The study showed that the chelating ligand, CMCR, was coordinated selectively to Ti(III) at eight coordination sites involving the oxygen atoms at the interface of the membrane/solution. The membrane was prepared, based on CMCR as an ionophore, sodium tetrakis(4-fluorophenyl) borate (NaTFPB) as a lipophilic ionic additive, and dioctylphthalate (DOP) as a plasticizer. The immobilization of the ionophore and surface characterization studies revealed that the performance of CMCR-immobilized PVC was equivalent to that of mobile ionophores in supported liquid membranes (SLMs). The strengths of the ion-ionophore (CMCR-Ti(OH)(OH_2_)_5_^2+^) interactions and the role of ionophores on membranes were studied via UV-Vis, Fourier transform infrared spectroscopy (FT–IR), scanning electron microscopy (SEM) and and X-ray diffraction (XRD).

## 1. Introduction

**Figure 1.**
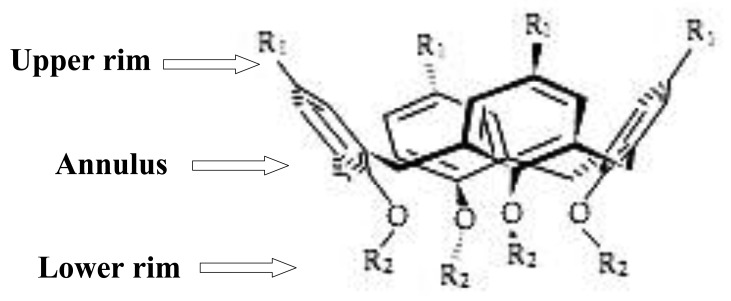
Division of calix[4]arene (applicable to all the calixarenes).

Calixarenes, as nano-baskets, are considered an important group of macrocyclic compounds and are the third most well-known host molecules after cyclodextrins and crown ethers. These macrocyclic molecules are a class of cyclooligomers that form after phenol-formaldehyde condensation, with defined upper and lower rims and a central annulus (Figure 1) [1]. Many researchers have focused on the study of calixarenes as electrode ionophores and optical and electrochemical sensors in recent decades.

Owing to the presence of cavities in structural calixarenes, they are able to act as host molecules. The “crater” or “basket” shape of calixarene plays a very important role in shaping the entire architecture of calixarene, due to its function in host-guest chemistry.

**Figure 2.**
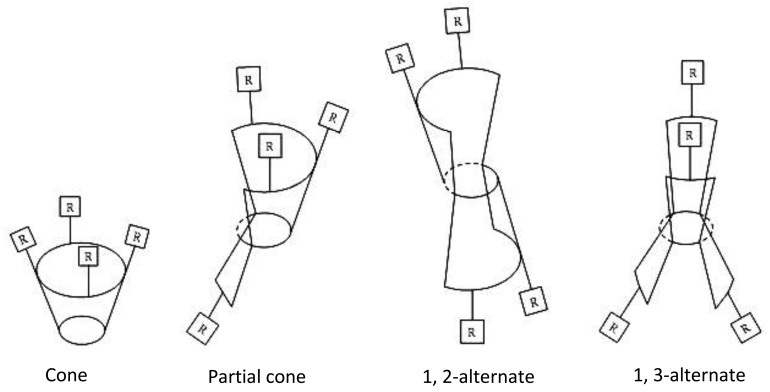
Conformers of cone shapes of calix[4]arenas.

Due to the presence of aromatic groups and the rotation of Ar-CH_2_-Ar bonds, calixarenes can assume different flexible forms. They came into existence as cone, partial cone, 1,2-alternate, and 1,3-alternate conformations (Figure 2), which were first suggested by Cornforth and later designated by Gutsche [2]. The mentioned structure of calixarenes and the character of other functional groups bonded to the basic skeleton of calixarenes leads to the interaction between these ligands and cations as well as interaction between anions and neutral species. These properties of calixarenes allow ionophores to use them for fabrication of ion-selective potentiometric sensors [3,4].

The CMCR ionophore as a basic component is responsible for the potential response of ion selective electrodes (ISEs) and has been used to prepare the membranes of these devices. As was previously indicated using the conductometric method, the CMCR ionophore forms 1:1 complexes with titanium (III) cations [5,6].

**Figure 3.**
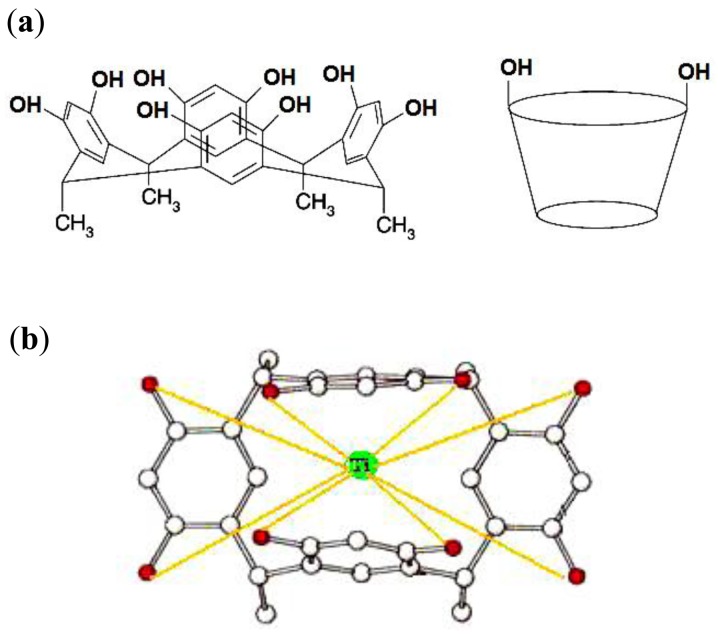
Optimal conformation of CMCR before (**a**) and after (**b**) complexation with Ti(OH)(OH_2_)_5_^2+^.

The results obtained from the UV-visible spectra confirm these observations. The optimized structures of the free CMCR (a) and CMCR-Ti(OH)(OH_2_)_5_^2+^ complex (b) are shown in Figure 3. In the complexed forms, the benzo groups are not coplanar due to the OH group in the Ti(OH)(OH_2_)_5_^2+^ cation, which is far enough from the O groups in CMCR to minimize the possible intermolecular repulsive force [7–10]. In the case of the CMCR ligand complex with Ti(OH)(OH_2_)_5_^2+^, the configuration of the complex will change from *cis* to *trans*, and a suitable binding interaction between OH in the CMCR ligand and the Ti(III) cation will occur. The contribution of solvation–desolvation energies of the central titanium cation in both the aqueous and the membrane phase for proper binding interaction between ligand (CMCR) and titanium (III) and the matching size of the ionic radius with the hole diameter of the ligand cannot be ignored.

Titanium is present in aqueous solution as: Ti(OH)_6_^3+^, Ti(OH)(OH_2_)_5_^2+^, Ti_2_O(OH_2_)_10_^4+^, Ti(OH)_3+*_x_*_ (*x* corresponding to the oxidation rate) and TiO_2_ [11]. The dissolution and hydrolysis of titanium chloride (III) (that was used as the source of titanium) is a multivariate process. The existence of each of these species or others is dependent on different variables such as pH, temperature, titanium concentration and hydrochloric acid concentration [12]. The Ti(OH)(OH_2_)_5_^2+^ cation prevails in a basic or slightly acidic solution, while other species are dominant in different pH of titanium aqueous solution [12,13].

$$\begin{array}{l} \;\text{Ti}{{\left(\text{OH}\right)}_{6}}^{3+}↔\text{Ti}(\text{OH}){{\left(\text{O}{\text{H}}_{2}\right)}_{5}}^{2+}↔{\text{Ti}}_{2}\text{O}{{\left({\text{OH}}_{2}\right)}_{10}}^{4+}↔\text{Ti}{\left(\text{OH}\right)}_{{3+}_{\text{x}}}\\ \text{pH}\;<1\;1–3\;3\;3< \end{array}$$

All of the measured solutions were in a pH in the range of 1–3 and we expect that in the crystal structure of titanium chloride(III), the dominant titanium species would be in the form of the Ti(OH)(OH_2_)_5_^2+^, a finding further supported by the literature.

In this study poly(vinyl chloride) (PVC)-based membranes incorporating CMCR as an ionophore have been prepared and explored as a titanium(III) selective sensor [14]. The strengths of the ion-ionophore (Ti(OH)(OH_2_)_5_^2+^-CMCR) interactions and the role of the ionophore on the membrane were also studied via UV-Vis, Fourier transform infrared spectroscopy (FT-IR), scanning electron microscopy (SEM) and X-ray diffraction (XRD).

## 2. Experimental

### 2.1. Apparatus

The Fourier transform infrared (FT-IR) spectra of the samples were recorded with a Perkin-Elmer FT-IR spectrophotometer (model Spectrum 100 series) using KBr disks. For UV-Vis spectroscopy, a Perkin-Elmer Lambda spectrophotometer 1650 PC (Shimadzu) was used. Scanning electron microscopy (SEM) techniques, using a Jeol scanning electron microscope (Model 1455 LEO) were used to study the immobilization of ionophore. X-ray diffraction (XRD) scans on a Shimadzu XRD-6000 Lab X wide-angle diffractometer were taken to characterize the membrane samples.

### 2.2. Reagents

Deionized bi-distilled water was used to prepare the solutions. The CMCR ionophore, a TiCl_3_ solution of about 15% was purchased from Merck. Polyvinyl chloride powder of high molecular weight (PVC, Fluka) and tetrahydrofurane (THF, Fluka) were used without further purification. Dioctylphthalate (DOP, Merck), sodium tetrakis (4-fluorophenyl) borate (NaTFPB, Fluka) were used, as a plasticizer and lipophilic anionic additive, respectively.

### 2.3. Membranes Preparation

The PVC-based membrane was prepared by dissolving appropriate amounts of ionophore (CMCR, 8 mg), anion excluder (NaTFPB, 3 mg), plasticizer (DOP, 59 mg) and polymeric matrix (PVC, 30 mg) in THF (3 mL). After complete dissolving of all components and mixing, a Pyrex tube was dipped in the solution obtained, and a 0.3 mm thick membrane formed. The tube was then pulled out from the mixture and kept at room temperature for 24 h. After preparation of the membrane, it was conditioned by soaking it in a 1.0 × 10^−3^ mol·L^−1^ TiCl_3_ solution for about 24 h.

### 2.4. Experimental Procedures

The membrane ISE, based on CMCR, is characterized using a number of techniques, including UV-Vis spectroscopy, Fourier transform infrared spectroscopy (FT-IR), scanning electron microscopy (SEM) and X-ray diffraction (XRD). FT-IR was used to characterize the interaction of titanium chloride (III) with the CMCR ionophore in a composite membrane. The absorption (or transmission) spectra of the molecular species samples were recorded in the 400 to 4,000 cm^−1^ wavenumber range using the KBr pellet technique. UV-Vis spectroscopy was used for the Ti(OH)(OH_2_)_5_^2+^-CMCR complexation study. Three sample solutions were prepared (1 × 10^−4^ M solution of CMCR in the absence and presence of 1 × 10^−4^ M TiCl_3_) in a binary mixture of acetonitrile–water. The absorption spectra of the solutions were then recorded for each sample in the range of 190 to 400 nm. For the SEM study the sample holders were cleaned and then dried with acetone solvent. The sample membranes were then fixed onto the holders and coated with gold in the vacuum chamber to increase the conductance of their surfaces. These samples were used to study the morphology of the membrane surfaces by SEM use. To characterize the membrane samples by X-ray diffraction the optimized membrane based on CMCR was prepared and stocked in the TiCl_3_ solutions for 24 h. Then the X-ray diffraction scans were taken on the membrane samples at a rate of two degrees per minute. Samples for XRD observations were mounted on an aluminium sample holder.

## 3. Results and Discussion

UV-Vis spectroscopy was used to investigate the interaction between the CMCR ionophore and the Ti(OH)(OH_2_)_5_^2+^ cation. In order to study the selective interaction of the CMCR as a potential ionophore, the UV-Vis spectra of the complex were obtained in the absence and presence of this cation in dry acetonitrile solution. According to the Hofmeister series (lyotropic series—favourable ability of the cations to complexation) for cations, significant interaction between CMCR with Ti(OH)(OH_2_)_5_^2+^ was expected [15].

**Figure 4.**
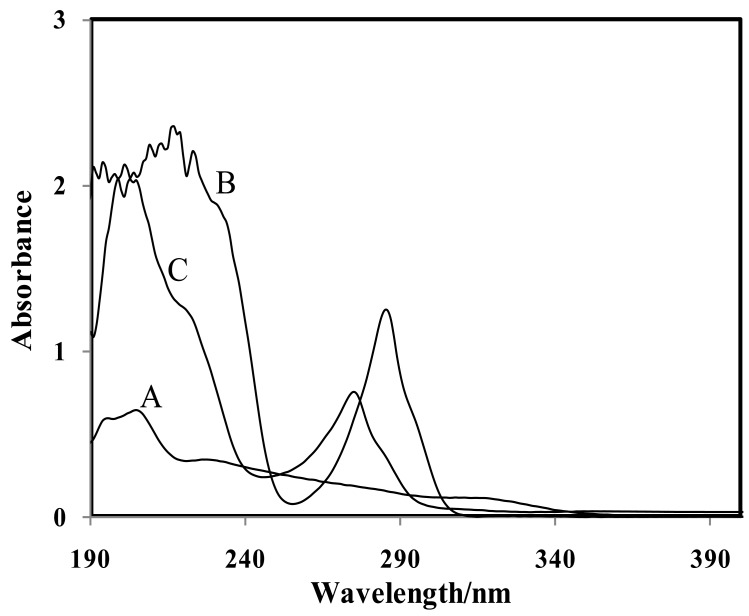
(**A**) UV-Vis absorption spectra of acetonitrile solutions of 1.0 × 10^−4^ M TiCl_3_; (**B**) 1.0 × 10^−4^ M CMCR in the absence of TiCl_3_; (C) CMCR 1.0 × 10^−4^ M treated with 1.0 × 10^−4^ M TiCl_3_ solution.

As shown in Figure 4, the results support this. From this figure it is obvious that the addition of ionophores (with two absorption maxima at 217 and 286 nm for CMCR) to an equilibrium solution of Ti(OH)(OH_2_)_5_^2+^ (with two absorption maxima at 196 and 205 nm) resulted in a simultaneous distinct decrease in absorption bands with an intensive wavelength shift from 196 to 274 nm for CMCR. These observations revealed that CMCR has a special tendency towards the Ti(OH)(OH_2_)_5_^2+^ cation.

**Figure 5.**
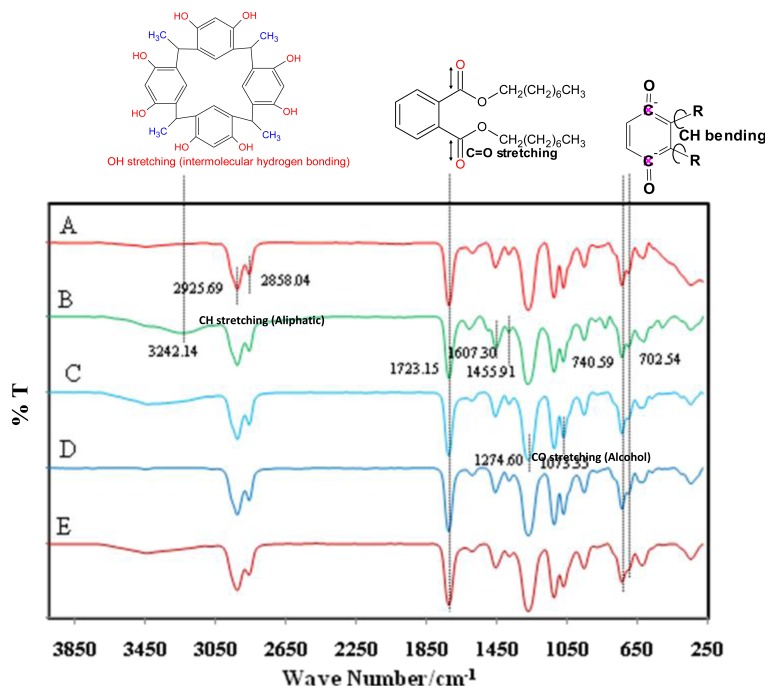
The FT-IR spectra of the membrane PVC sensor based on CMCR ionophore in different states: (**A**) blank membrane, before (**B**), after (**C**) destocking in 1.0 × 10^−3^ M TiCl_3_ solution for 24 h, (**D**) stocked in 1.0 × 10^−2^ M TiCl_3_ solution for 24 h and (**E**) two months.

The influence of ionophores, titanium cations of the proposed membrane sensors, was investigated by FT-IR spectroscopy. Figure 5 shows the characteristic peaks of FT-IR for the titanium(III) membrane sensor based on the CMCR ionophore in five steps ((A) blank membrane, before (B), after (C) destocking in 1.0 × 10^−3^ M TiCl_3_ solution for 24 h, (D) stocked in 1.0 × 10^−2^ M TiCl_3_ solution for 24 h and (E) 2 months), respectively.

The presence of the Ti(OH)(OH_2_)_5_^2+^ cation caused the shift and an increase in the absorption intensity of the OH stretching at 3,242.14 cm^−1^ (spectrum C in Figure 5) in the Ti(II)-CMCR complex [16]. These displacements have been attributed to an O–H stretching band by Ti–O coordination [17]. Clearly, there is no peak related to the ionophores (OH stretching in the CMCR ring) in the blank membrane (spectrum A in Figure 5). The presence of DOP as plasticizer in the membrane compositions based on CMCR ionophore is confirmed by the strong absorption bands related to C−O stretching appear in the 1,100–1,300 cm^−1^ region and C=O stretching appears at 1,723 cm^−1^. Moreover, the peak due to C−H out of plane bending in 740 to 743 cm^−1^ region is a common absorption band for the DOP plasticizer. As it can be observed from all FT-IR spectra, the plasticizer (DOP) and ionophore (CMCR) show the common peak of C−H sp^3^ stretching in 2,858 cm^−1^. Besides, the common peak of =C−H sp^2^ stretching which appears at 2,925 cm^−1^ indicates the presence of plasticizers (DOP) and lipophilic additive (NaTFPB). It is noteworthy to mention that, due to a very low amount of lipophilic additives in the matrix of the membranes and high intensity of C=O, C=C absorption bands which have overlapped with the C−Br stretching, it is not possible to deduce the presence of lipophilic additives individually from the presented FT-IR spectra.

The changes in the concentration of solutions of the titanium cation from 1.0 × 10^−3^ M (spectrum C) to 1.0 × 10^−2^ M (spectrum D) did not cause any significant difference in the spectra of the two membrane sensors. Consequently, the activity of the internal solution for the fabricated membrane electrodes based on CMCR can be kept at 1.0 × 10^−3^ M for TiCl_3_ solutions. After 2 months, no significant changes were seen in the spectra. Therefore, these membrane electrodes can be used successfully without any major decrease in response.

**Figure 6.**
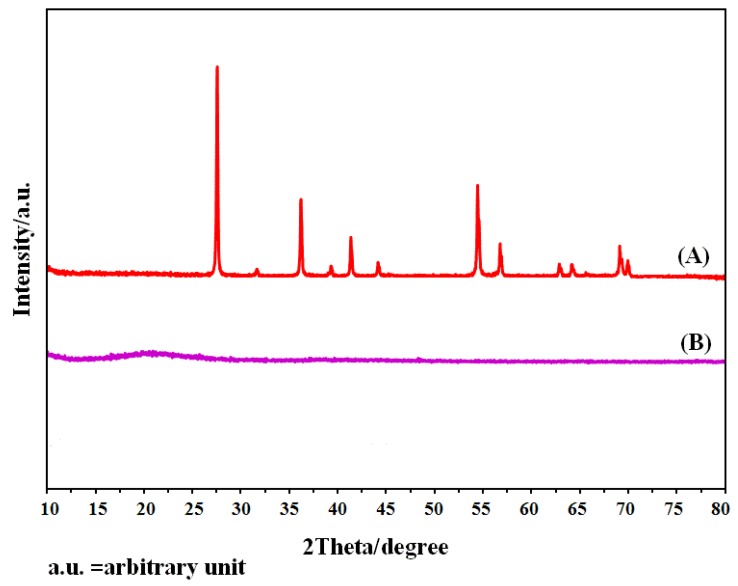
X-ray diffraction patterns of (**A**) pure TiO_2_, (**B**) membrane based CMCR stocked in 1.0 × 10^−2^ M TiCl_3_ solution.

X-ray diffraction (XRD) facilitates understanding of the physical properties of metals, polymeric materials, and other solids. When an X-ray beam strikes a crystal, a portion of this beam is scattered by the layer of atoms at the surface. If the distances between the scattering centers are of the same order of magnitude as the wavelength of the radiation, then interference take place among the scattering rays, resulting in diffraction. Figure 6 shows the X-ray diffraction patterns of the optimized polymeric membrane based on the CMCR ionophore after storing for 24 h in 1.0 × 10^−2^ M TiCl_3_. The XRD patterns of the fabricated membrane based on CMCR (pattern B) didn't indicate any crystalline character. These observations evidence the amorphous structures of the fabricated membranes without any crystalline structure due to the presence of TiO_2_ (pattern A).

**Figure 7.**
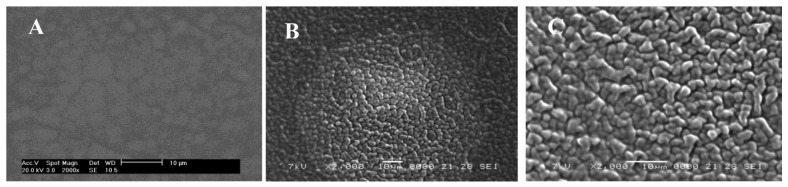
Scanning electron microscopy micrographs of the surfaces of the CMCR-immobilized membranes at the magnification of 2,000× (**A**) without ionophore, (**B**) with fresh membrane, and (**C**) with membrane employed for two months.

Figure 7 shows the surface morphology of the fabricated membrane based on CMCR as investigated by scanning electron microscopy (SEM). The PVC-membrane without the CMCR ionophore exhibited a physically tight structure, as illustrated in Figure 7(A), while the membranes with the CMCR ionophore exhibited a surface with a loose and permeable structure that included channels to diffuse the Ti(III) cations (B). Moreover, after two months, the surface morphologies for the two membranes showed a swollen structure without any leakage (C).

## 4. Conclusions

In this study, UV-Vis, FT-IR, SEM and XRD analyses supported the assertion that interaction occurred between a CMCR ligand and titanium (III) ions in the membrane phase. The results demonstrate that the application of CMCR in the polymeric membrane phase as a selective ionophore creates a cation sensor for the quantification of titanium (III) cations. Accordingly, we can use this ligand as a sensing element in the membrane in order to fabricate an ion selective electrode.
